# Association between Thyroid Cancer and Weight Change: A Longitudinal Follow-Up Study

**DOI:** 10.3390/ijerph19116753

**Published:** 2022-05-31

**Authors:** Young Ju Jin, Jeong Hun Hah, Mi Jung Kwon, Ji Hee Kim, Joo-Hee Kim, Sung-Kyun Kim, Bumjung Park, Hyo Geun Choi

**Affiliations:** 1 Department of Otorhinolaryngology—Head & Neck Surgery, Wonkwang University Hospital, Wonkwang University College of Medicine, Iksan 54538, Korea; chindol1@wku.ac.kr; 2 ThanQ Seoul Thyroid—Head and Neck Surgery Center, Seoul 06150, Korea; jhunhah@gmail.com; 3 Department of Pathology, Hallym University Sacred Heart Hospital, Hallym University College of Medicine, Anyang 14068, Korea; mulank99@hallym.or.kr; 4 Department of Neurosurgery, Hallym University College of Medicine, Anyang 14068, Korea; kimjihee@hallym.or.kr; 5 Division of Pulmonary, Allergy, and Critical Care Medicine, Department of Medicine, Hallym University Sacred Heart Hospital, Hallym University College of Medicine, Anyang 14068, Korea; luxjhee@hallym.or.kr; 6 Department of Otorhinolaryngology—Head & Neck Surgery, Hallym University College of Medicine, Dongtan 18450, Korea; madein811022@gmail.com; 7 Department of Otorhinolaryngology—Head & Neck Surgery, Hallym University Sacred Heart Hospital, Hallym University College of Medicine, Anyang 14068, Korea; bumjung426@gmail.com; 8 Hallym Data Science Laboratory, Hallym University College of Medicine, Anyang 14068, Korea

**Keywords:** thyroid cancer, thyroidectomy, body weight change, hypertension, obesity

## Abstract

Background: The purpose of this study was to evaluate body mass index (BMI) and systolic blood pressure (SBP)/diastolic blood pressure (DBP) between Korean adults who underwent thyroidectomy and comparison groups. Methods: Data were included from the Korean National Health Insurance Service-Health Screening Cohort (2002–2015). BMI and SBP/DBP were measured before thyroidectomy, 1 and 2 years after thyroidectomy (*n* = 1995 in study I, *n* = 2162 in study II), comparing 1:4 matched participants (*n* = 7980 in study I, *n* = 8648 in study II). The paired t-test and linear mixed model were used to identify the differences between groups. Results: DBP in both thyroid cancer II and comparison II group were significantly lower after thyroidectomy than before thyroidectomy. However, the interaction effect of thyroidectomy in study I and study II did not reach statistical significance. Conclusion: BMI, SBP and DBP were not significantly different between the thyroidectomy group and the matched comparison group.

## 1. Introduction

Obesity and hypertension are preventable and modifiable risk factors for cardiovascular disease, stroke, type 2 diabetes mellitus, Alzheimer’s disease and so on [1,2]. The prevalence of overweight and obesity in adulthood has increased by 27% in recent decades [3]. The diagnosis of obesity is based on body mass index (BMI), estimated as weight in kilograms divided by height in meters squared (kg/m^2^). BMI is considered overweight over 25 and over 30 is considered obese [4]. Hypertension, defined as a systolic blood pressure of 140 mmHg or higher and/or diastolic blood pressure 90 mmHg or higher, affected approximately 31.1% of adults worldwide in 2010 [2]. Obesity and hypertension can be caused by common factors, such as unhealthy diet, decreased physical activity, low energy expenditure, genetics, aging and so on [2,5].

Thyroid cancer is the third most common malignancy in women, and the overall incidence has increased by 3% annually in the past several decades in the US [6]. For treatment, the American Thyroid Association (ATA) guidelines suggest near total or total thyroidectomy in advanced cases and postoperative radioiodine ablative therapy according to the disease status [7]. Changes in thyroid hormone levels after thyroidectomy can lead to obesity through alterations in the basal metabolic rate [8]. In general, people with hyperthyroidism lose weight due to a high basal metabolic rate, and people with hypothyroidism gain weight due to a low basal metabolic rate [9]. Post-thyroidectomy weight gain is a main concern and cause of dissatisfaction in both hyperthyroidism and hypothyroidism patients [10,11,12,13]. However, there are only few studies about weight change after thyroidectomy in thyroid cancer participants. Some studies have reported weight gain after thyroidectomy in the thyroid cancer group [10,13]. Others reported that patients showed no weight change after thyroidectomy, although subclinical hyperthyroidism status could be expected to lead to weight loss [14,15].

An imbalance in thyroid hormone levels after thyroidectomy could cause hypertension by altering cardiac output, systemic vascular resistance and arterial stiffness [16]. Weight change could be aggravated by iatrogenic hyper or hypothyroidism after taking levothyroxine replacement and decreased leptin levels during thyroid hormone withdrawal [9,17]. Moreover, obesity itself is a major cause of hypertension. Naturally, body weight increases by 0.35 to 0.83 kg annually in females and 0.29 to 0.4 kg annually in males in various populations [11]. Although there is a possibility that there is a relationship between thyroid cancer and weight change or thyroid cancer and blood pressure change, existing reports are rare.

The purpose of this study was to investigate the changes in body mass index (BMI) and systolic blood pressure (SBP)/diastolic blood pressure (DBP) in Korean adults who underwent thyroidectomy and compare the changes to those observed in the comparison groups.

## 2. Materials and Methods

### 2.1. Study Population

This study was approved by the ethics committee of Hallym University (2019-10-023). The Institutional Review Board waived the requirement for written informed consent. A detailed description of the Korean National Health Insurance Service-Health Screening Cohort data is provided elsewhere [18].

### 2.2. Definition of Thyroid Cancer (Independent Variable)

Thyroid cancer was assigned as a diagnosis with ICD-10 code C73 (malignant neoplasm of thyroid gland) treated by any type of thyroidectomy (P4551, P4552, P4553, P4554, and P4561), based on the criteria mentioned in our previous studies [18,19]. 

### 2.3. Definition of Weight Change (Dependent Variable)

The definition of weight change after 1 year was the BMI change between the latest measured BMI before thyroid cancer diagnosis and the BMI in the first year after thyroid cancer diagnosis (Study I). Moreover, the definition of weight change after 2 years was the BMI change between the latest BMI before thyroid cancer diagnosis and the BMI in the second year after thyroid cancer diagnosis (Study II).

### 2.4. Definition of Blood Pressure Change (Dependent Variable)

Blood pressure change after 1 year was assigned as the difference in blood pressure between the latest blood pressure measured before thyroid cancer diagnosis and blood pressure measured in the first year after thyroid cancer diagnosis (Study I). Blood pressure change after 2 years was assigned as the difference in blood pressure between the latest blood pressure measured before thyroid cancer diagnosis and blood pressure measured in the second year after thyroid cancer diagnosis (Study II).

### 2.5. Participant Selection

Among 514,866 participants, thyroid cancer participants were selected using 615,488,428 medical claim codes from 2002 to 2015 (*n* = 5536), and excluded those who did not have follow-up data in both the first year and second year (*n* = 1826). Comparison participants were included in cases where thyroid cancer was confirmed without thyroidectomy history or not diagnosed with thyroid cancer from 2002 to 2015 (*n* = 509,330). Among 509,330 comparison participants, 2287 participants were excluded because they were diagnosed with thyroid cancer without thyroidectomy history. Because all participants did not have this health check-up annually, some thyroid cancer participants had follow-up records after the first year (*n* = 2068, thyroid cancer I), and other thyroid cancer participants had follow-up records after the second year after thyroid cancer diagnosis (*n* = 2226, thyroid cancer II). In total, 584 thyroid cancer participants had data from both first- and second-year follow-ups.

**Figure 1:**
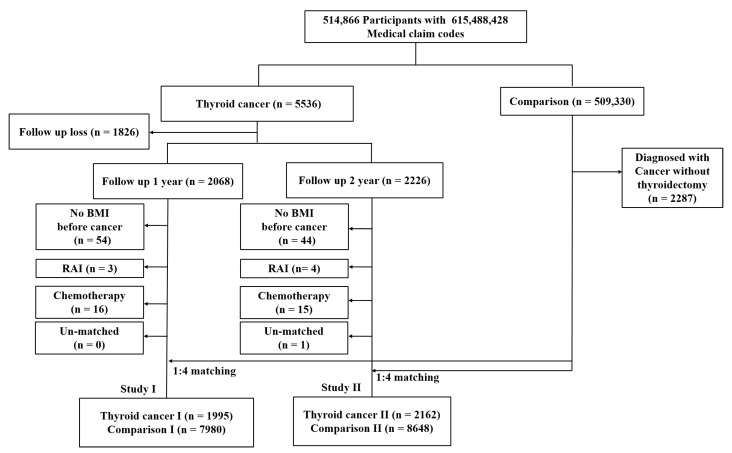
A schematic illustration of the participant selection process that was used in the present study. Of a total of 514,866 participants, 5536 thyroid cancer participants and 509,330 comparison participants were included in this study. Of a total of 514,866 participants, 1995 thyroid cancer I participants were 1:4 matched with 7980 comparison I participants for age, sex, income, region of residence and obesity. Of a total of 514,866 participants, 2162 thyroid cancer II participants were 1:4 matched with 8648 comparison II participants for age, sex, income, region of residence and obesity.

In study I, 54 thyroid cancer participants were excluded due to no record of BMI before thyroid cancer diagnosis. Thyroid cancer participants with a history of radioactive therapy (*n* = 3) and chemotherapy (*n* = 16) were excluded. Thyroid cancer participants and comparison participants were 1:4 matched for age, sex, income, region of residence and obesity. To reduce selection bias, a random number order was used to select the comparison participants. The index date of each thyroid cancer participant was adjusted as the time of treatment of thyroid cancer. The index date of the comparison participants was adjusted as the index date of the matched thyroid cancer participants. Therefore, each matched thyroid cancer participant and comparison participants had the same index date. Through the matching procedure, 499,063 comparison participants were excluded. Finally, 1995 thyroid cancer I participants were 1:4 matched with 7980 comparison I participants (Figure 1).

In study II, thyroid cancer II participants without BMI records before thyroid cancer diagnosis (*n* = 44) were excluded. Thyroid cancer participants with a history of radioactive therapy (*n* = 4) or chemotherapy (*n* = 15) were excluded. Thyroid cancer participants and comparison participants were 1:4 matched for age, sex, income, region of residence and obesity. To reduce selection bias, a random number order was used to select the comparison participants. The index date of each thyroid cancer participant was adjusted as the time of treatment of thyroid cancer. The index date of the comparison participants was adjusted as the index date of the matched thyroid cancer participants. Therefore, each thyroid cancer participant and the matched comparison participants had the same index date. Through the matching procedure, 1 thyroid cancer participant and 498,575 comparison participants were excluded. Finally, 2162 thyroid cancer II participants were 1:4 matched with 8648 comparison II participants (Figure 1).

### 2.6. Covariates

The participants were divided into age groups with 5-year intervals of 40–44, 45–49, 50–54 …, and 80–84 years old, resulting in total of 9 age groups. Income was graded into 5 classes (class 1 (lowest income)-5 (highest income)). The residence was divided into either of urban or rural areas.

Tobacco smoking, alcohol consumption and obesity were categorized based on BMI (kg/m^2^), and fasting blood glucose and total cholesterol were measured. Missing fasting blood glucose (3/9975 (0.030%) in study I, 8/10,810 (0.074%)) in study II) and total cholesterol (5/9975 (0.050%) in study I) values were replaced with mean values of the variable from the data of the final selected participants. The Charlson comorbidity index (CCI), excluding thyroid cancer, was measured as a continuous variable (0 (no comorbidities) to 29 (multiple comorbidities)) [20].

### 2.7. Statistical Analyses

The general characteristics of the population were compared between the thyroid cancer and comparison groups. The categorical variables were analyzed using the chi-square test, while the continuous variables were compared using the independent *t*-test. 

Using the linear mixed model, we calculated the weight/SBP/DBP changes between the thyroid and comparison group (S1 description).

For the subgroup analyses, we divided participants by age and sex (<55 years old and ≥55 years old; men and women) and by obesity status (underweight, normal, overweight, obese I and obese II).

Two-tailed analyses were performed, and a *p*-value of less than 0.05/3 was regarded as significant (Bonferroni correction). SAS version 9.4 (SAS Institute Inc., Cary, NC, USA) was used for statistical analyses.

## 3. Results

### Detailed Descriptions

Age, sex, income, region of residence and obesity were exactly matched between the thyroid cancer and comparison groups in studies I and II (all *p* = 1.000). Both smoking status and CCI score were significantly different between the thyroid cancer I and comparison I groups and thyroid cancer II and comparison II groups, respectively (Table 1).

The mean values of BMI and SBP/DBP were not different between the thyroid cancer I and comparison I groups. The interaction effect of thyroidectomy on BMI/SBP/DBP in all participants did not show statistical significance in study I (Table 2).

The mean value of DBP decreased in the thyroid cancer II group after thyroidectomy, and it decreased in comparison II group after 2 years compared with the mean value from the previous index dates. However, the interaction effects did not show statistical significance in study II (Table 3).

In the subgroup analyses according to age, sex, and BMI (Table 2, Table 3, Tables S2 and S3), BMI and SBP/DBP significantly changed in some subgroups. However, none of them showed effects of the thyroidectomy–time interaction in either study I or II.

## 4. Discussion

In this study, we could not find any significant differences between the thyroidectomy group and the 1:4 matched comparison group in the changes in BMI, SBP or DBP over time using the interaction model comparison.

Previously, there have been concerns about body weight changes after thyroidectomy. Most studies have demonstrated postoperative weight gain in patients with various thyroid-related diseases. A retrospective review of 267 thyroidectomy patients with various diseases showed that the mean preintervention body weight significantly increased from 70.8 (SD = 16.0) to 72.5 (SD = 16.4) kg at 9 months after the operation and that post-operation TSH levels were not related to the subsequent weight changes [12]. In another study, 120 hypothyroidism patients treated with thyroid hormone replacement after thyroidectomy were compared with three matched groups, pre-existing hypothyroidism, no thyroid disease and thyroid cancer. Postsurgical hypothyroidism patients gained 3.1 kg at 1 year after the operation, whereas pre-existing hypothyroidism patients who did not undergo an operation gained 2.2 kg during the same time period. All patients with hypothyroidism experienced more weight gain than those without hypothyroidism, even though they maintained euthyroidism with treatment [11]. Moreover, significant weight gain has been reported in hyperthyroidism patients after treatment [21,22,23]. A meta-analysis of weight change after thyroidectomy revealed that patients with thyroidectomy experience weight gain. The values of body weight increased following indication for total thyroidectomy by 1.3 kg in the thyroid cancer group, 1.55 kg in the euthyroid (goiter) group and 5.19 kg in the hyperthyroidism group [10]. Polotsky et al. estimated weight change in 153 thyroid cancer patients and found that body weight increased by 3.2% over 3–5 years [13]. Meanwhile, there are some studies that have revealed no weight gain after thyroidectomy. Glick et al. reported no weight gain or unpredictable weight changes in an analysis of 107 total thyroidectomy patients who underwent the operation for a wide spectrum of pathologies. The mean body weight change did not show a significant difference when considering preoperative thyroid function status (0.06 (SD = 6.9) kg, *p* = 0.094) [24]. Weinreb et al. analyzed 102 patients who underwent total thyroidectomy for thyroid cancer and 92 comparisons with thyroid nodules or goiter. There was no significant weight gain over time (mean follow-up duration: 7.6 years) in the thyroid cancer group compared to the comparison group [15]. Kedia et al. performed a retrospective study of 291 patients who underwent total thyroidectomy for thyroid cancer (147 participants) and benign disease (144 participants). There were no weight differences between the two groups at years 1 through 3 [14].

There are several possible causes of weight change after treatment for thyroid cancer. First, taking suppressive doses of levothyroxine can lead to subclinical hyperthyroidism, which can lead to weight loss or a lack of weight gain. Serum thyroid-stimulating hormone (TSH) levels are positively associated with body weight. After undergoing thyroidectomy as a treatment for thyroid cancer, patients took levothyroxine to replace thyroid hormone and to suppress TSH. Hyperthyroidism frequently presents with weight loss [8]. However, similar to our results, some previous studies did not identify significant weight change in subclinical hyperthyroidism patients. Therefore, there could be other unknown factors for body weight changes. Second, body weight and energy expenditure are mainly regulated by two hormones, leptin and ghrelin. Leptin, released by adipose tissue, is positively associated with BMI. Ghrelin, secreted by the stomach, is negatively correlated with BMI in humans [25]. Lin et al. suggested that during thyroid hormone withdrawal, leptin levels were decreased, while adiponectin levels were not changed [17]. Although there is a possibility of an association between leptin level and weight change in thyroid cancer participants, this remains to be further studied.

In our study, DBP increased in the thyroid cancer II group and decreased in the comparison II group, but not in the thyroid cancer I or comparison I group. In all groups, the interaction effects between the thyroid cancer group and the comparison group did not reach statistical significance. SBP did not reveal a significant association. Theoretically, thyroidectomy could influence blood pressure changes by dysregulating thyroid hormone. Hyperthyroidism could induce hypertension via increased cardiac output, positive chronotropic effect, decreased systemic vascular resistance, increased arterial stiffness and increased blood volume. Hypothyroidism could also cause hypertension by decreasing cardiac output, exerting negative chronotropic effects and increasing arterial stiffness and systemic vascular resistance, which are aggravated by hypercholesterolemia, low-grade inflammation and endothelial dysfunction in peripheral arteries [16]. However, previous studies on the association between thyroidectomy and blood pressure changes are limited. In a previous study, 24 women after total or near total thyroidectomy for differentiated thyroid cancer were evaluated for aortic stiffness, which is known as an independent predictor of cardiac events. The investigators found increased aortic stiffness and decreased diastolic cardiac function in both hypothyroidism (L-T4 withdrawal) and subclinical hyperthyroidism (L-T4 therapy) patients, compared to the respective characteristics in comparisons (22 healthy participants matched for age and sex) [26]. In a review article, multikinase inhibitors used for untreatable thyroid cancer could have adverse effects on hypertension [27]. However, they were compared with the matched comparison group. As few studies have evaluated this relationship in thyroid cancer, we could not find further references.

There are several limitations of this study. First, we included patients who underwent lobectomy or total thyroidectomy. Remnant thyroid function and hormone replacement treatment could differ according to the extent of surgery. However, in a retrospective study with 967 patients with thyroid cancer, changes between total thyroidectomy and lobectomy patients were compared. Weight change did not differ between total thyroidectomy and thyroid lobectomy patients [28]. Second, we did not have information about thyroid function tests, including TSH, free T4 and T3, which have important relationships with body weight. This study has several strengths. First, we selected participants from a large representative national health screening cohort. Therefore, this report is the largest study to show that in participants who had undergone total thyroidectomy or lobectomy for thyroid cancer, BMI in over 55 year old women and DBP in over 55 year old men and women were decreased significantly from pre- to post-operation in the thyroid cancer II group. Second, this study is the first report comparing BMI, SBP and DBP between thyroid cancer participants and comparison participants, who were 1:4 matched for age, sex, income, region of residence and obesity. The two groups did not show significant differences in BMI, SBP or DBP.

## 5. Conclusions

The mean value of BMI, SBP and DBP were not significantly different between the thyroidectomy group with thyroid cancer and the 1:4 matched comparison group after 1 or 2 years.

## Figures and Tables

**Table 1:** General characteristics of participants.

Characteristics		Study I			Study II		
		Thyroid Cancer I	Comparison I	*p*-Value	Thyroid Cancer II	Comparison II	*p*-Value
Age (years old, *n*, %)				1.000			1.000
	40–44	29 (1.5)	116 (1.5)		46 (2.1)	184 (2.1)	
	45–49	345 (17.3)	1380 (17.3)		328 (15.2)	1312 (15.2)	
	50–54	593 (29.7)	2372 (29.7)		748 (34.6)	2992 (34.6)	
	55–59	559 (28.0)	2236 (28.0)		482 (22.3)	1928 (22.3)	
	60–64	224 (11.2)	896 (11.2)		287 (13.3)	1148 (13.3)	
	65–69	154 (7.7)	616 (7.7)		139 (6.4)	556 (6.4)	
	70–74	63 (3.2)	252 (3.2)		109 (5.0)	436 (5.0)	
	75–79	26 (1.3)	104 (1.3)		18 (0.8)	72 (0.8)	
	80–84	2 (0.1)	8 (0.1)		5 (0.2)	20 (0.2)	
Sex (*n*, %)				1.000			1.000
	Male	512 (25.7)	2048 (25.7)		510 (23.6)	2040 (23.6)	
	Female	1483 (74.3)	5932 (74.3)		1652 (76.4)	6608 (76.4)	
Income (*n*, %)				1.000			1.000
	1 (lowest)	262 (13.1)	1048 (13.1)		257 (11.9)	1028 (11.9)	
	2	284 (14.2)	1136 (14.2)		248 (11.5)	992 (11.5)	
	3	314 (15.7)	1256 (15.7)		344 (15.9)	1376 (15.9)	
	4	338 (16.9)	1352 (16.9)		430 (19.9)	1720 (19.9)	
	5 (highest)	797 (40.0)	3188 (40.0)		883 (40.8)	3532 (40.8)	
Region of residence (*n*, %)				1.000			1.000
	Urban	953 (47.8)	3812 (47.8)		1058 (48.9)	4232 (48.9)	
	Rural	1042 (52.2)	4168 (52.2)		1104 (51.1)	4416 (51.1)	
Obesity ^‡^				1.000			1.000
	Underweight	20 (1.0)	80 (1.0)		31 (1.4)	124 (1.4)	
	Normal	668 (33.5)	2672 (33.5)		725 (33.5)	2900 (33.5)	
	Overweight	571 (28.6)	2284 (28.6)		631 (29.2)	2524 (29.2)	
	Obese I	662 (33.2)	2648 (33.2)		687 (31.8)	2748 (31.8)	
	Obese II	74 (3.7)	296 (3.7)		88 (4.1)	352 (4.1)	
Smoking status (*n*, %)				<0.001 *			0.018 *
	Nonsmoker	1712 (85.8)	6601 (82.7)		1872 (86.6)	7327 (84.7)	
	Past smoker	166 (8.3)	661 (8.3)		154 (7.1)	620 (7.2)	
	Current smoker	117 (5.9)	718 (9.0)		136 (6.3)	701 (8.1)	
Alcohol consumption (*n*, %)				0.124			0.102
	<1 time a week	1556 (78.0)	6094 (76.4)		1723 (79.7)	6752 (78.1)	
	≥1 time a week	439 (22.0)	1886 (23.6)		439 (20.3)	1896 (21.9)	
Fasting blood glucose (*n*, %)				0.447			0.078
	<100 mg/dL	1386 (69.5)	5508 (69.0)		1533 (70.9)	5936 (68.6)	
	100–125 mg/dL	497 (24.9)	1963 (24.6)		510 (23.6)	2151 (24.9)	
	≥126 mg/dL	112 (5.6)	509 (6.4)		119 (5.5)	561 (6.5)	
Total cholesterol (*n*, %)				0.059			0.002 *
	<200 mg/dL	1073 (53.8)	4059 (50.9)		1157 (53.5)	4263 (49.3)	
	200–239 mg/dL	657 (32.9)	2763 (34.6)		716 (33.1)	3066 (35.5)	
	≥240 mg/dL	265 (13.3)	1158 (14.5)		289 (13.4)	1319 (15.3)	
CCI score (*n*, %)				<0.001 *			<0.001 *
	0	1270 (63.7)	6440 (80.7)		1374 (63.6)	6988 (80.8)	
	1	314 (15.7)	916 (11.5)		336 (15.5)	998 (11.5)	
	2	94 (4.7)	334 (4.2)		127 (5.9)	357 (4.1)	
	3	36 (1.8)	144 (1.8)		35 (1.6)	151 (1.8)	
	≥4	281 (14.1)	146 (1.8)		290 (13.4)	154 (1.8)	
Systolic blood pressure (mean, SD)		123.95 ± 15.38	123.33 ± 15.90	0.116	123.77 ± 15.16	123.47 ± 15.78	0.430
Diastolic blood pressure (mean, SD)		77.38 ± 10.21	76.77 ± 10.51	0.019 ^†^	77.46 ± 10.06	76.86 ± 10.35	0.017 ^†^

Abbreviations: BMI, body mass index, kg/m^2^; CCI, Charlson comorbidity index; * chi-square test. Significance at *p* < 0.05; ^†^ independent *t*-test. Significance at *p* < 0.05; ^‡^ obesity (BMI, body mass index, kg/m^2^) was categorized as <18.5 (underweight), ≥18.5 to <23 (normal), ≥23 to <25 (overweight), ≥25 to <30 (obese I), and ≥30 (obese II).

**Table 2:** Difference in mean values of BMI and blood pressure between pre and 1-year post thyroid cancer in study I according to age and sex.

Characteristics		Thyroid Cancer I			Comparison I			Interaction ^‡^	Linear Mixed Model ^¶^	
		Previous (Mean, SD)	Post 1 yr (Mean, SD)	*p*-Value	Previous (Mean, SD)	Post 1 yr (Mean, SD)	*p*-Value	*p*-Value	EV ^§^	*p*-Value
Total participants (*n* = 9975)										
	BMI	24.26 ± 2.97	24.19 ± 3.05	0.020	24.22 ± 2.94	24.21 ± 2.94	0.262	0.245	0.029	0.691
	SBP	123.95 ± 15.38	123.45 ± 14.20	0.152	123.33 ± 15.90	123.36 ± 15.02	0.880	0.423	−0.299	0.214
	DBP	77.38 ± 10.21	76.88 ± 9.63	0.043	76.77 ± 10.51	76.57 ± 9.85	0.106	0.837	0.325	0.044 ^†^
Age < 55 years old, men (*n* = 1365)										
	BMI	24.57 ± 2.52	24.60 ± 2.65	0.656	24.60 ± 2.74	24.57 ± 2.64	0.488	0.598	−0.021	0.906
	SBP	124.15 ± 13.92	122.77 ± 13.29	0.127	125.20 ± 14.52	124.47 ± 14.65	0.104	0.172	−1.493	0.012 ^†^
	DBP	79.27 ± 9.92	78.86 ± 10.21	0.532	79.77 ± 10.11	79.03 ± 10.00	0.027	0.209	0.152	0.710
Age < 55 years old, women (*n* = 3470)										
	BMI	23.59 ± 2.93	23.58 ± 2.97	0.867	23.57 ± 2.79	23.62 ± 2.85	0.015 *	0.187	0.019	0.871
	SBP	120.19 ± 13.90	120.42 ± 14.26	0.688	119.18 ± 15.23	119.56 ± 14.13	0.176	0.868	0.152	0.688
	DBP	75.43 ± 9.85	75.52 ± 9.69	0.827	74.71 ± 10.50	74.90 ± 9.69	0.345	0.931	0.191	0.458
Age ≥ 55 years old, men (*n* = 1195)										
	BMI	24.95 ± 2.73	24.86 ± 2.67	0.296	24.96 ± 2.69	24.83 ± 2.70	<0.001 *	0.461	−0.002	0.990
	SBP	127.86 ± 14.79	126.24 ± 13.23	0.090	127.68 ± 15.50	127.66 ± 15.08	0.965	0.613	−1.112	0.127
	DBP	80.13 ± 9.37	78.50 ± 8.78	0.018	79.44 ± 10.30	78.92 ± 9.66	0.159	0.694	0.684	0.187
Age ≥ 55 years old, women (*n* = 3945)										
	BMI	24.52 ± 3.12	24.37 ± 3.26	0.003 *	24.45 ± 3.09	24.41 ± 3.11	0.069	0.119	0.065	0.594
	SBP	126.00 ± 16.55	125.51 ± 14.23	0.397	125.02 ± 16.27	125.01 ± 15.17	0.982	0.934	−0.041	0.921
	DBP	77.61 ± 10.53	76.89 ± 9.43	0.079	76.73 ± 10.28	76.48 ± 9.67	0.200	0.712	0.426	0.115

Abbreviations: BMI, body mass index, kg/m^2^; CCI, Charlson comorbidity index; EV, estimated value; SBP, systolic blood pressure; DBP, diastolic blood pressure; * Paired *t*-test, significance at *p* < 0.05/3; ^†^ Linear mixed model, significance at *p* < 0.05/3; ^‡^ Interaction effects between time and group; ^§^ Estimated value of linear mixed model for thyroid cancer I group based on the comparison I group; ^¶^ Fixed effects were age, sex, income, region of residence, thyroid cancer, and time of measurement. Random effects were BMI, systolic blood pressure, diastolic blood pressure, fasting blood glucose, total cholesterol, smoking, alcohol consumption, and CCI scores.

**Table 3:** Difference in mean values of BMI and blood pressure between pre and 2-year post thyroid cancer in study II according to age and sex.

Characteristics		Thyroid Cancer II			Comparison II			Interaction ^‡^	Linear Mixed Model ^¶^	
		Previous (Mean, SD)	Post 2 yr (Mean, SD)	*p*-Value	Previous (Mean, SD)	Post 2 yr (Mean, SD)	*p*-Value	*p*-Value	EV ^§^	*p*-Value
Total participants (*n* = 10,810)										
	BMI	24.22 ± 2.93	24.15 ± 3.00	0.016	24.23 ± 2.99	24.21 ± 3.03	0.130	0.300	−0.005	0.937
	SBP	123.77 ± 15.16	123.44 ± 14.36	0.338	123.47 ± 15.78	123.45 ± 15.26	0.934	0.978	−0.370	0.123
	DBP	77.46 ± 10.06	76.73 ± 9.34	0.002 *	76.86 ± 10.35	76.41 ± 9.91	<0.001 *	0.724	0.493	0.002 ^†^
Age < 55 years old, men (*n* = 1430)										
	BMI	24.81 ± 2.65	24.88 ± 2.85	0.328	24.85 ± 2.67	24.81 ± 2.70	0.219	0.151	−0.012	0.943
	SBP	124.16 ± 14.33	123.66 ± 13.13	0.557	125.36 ± 13.70	125.50 ± 14.33	0.750	0.259	−0.726	0.218
	DBP	80.03 ± 10.14	78.27 ± 9.55	0.005 *	79.69 ± 10.01	79.41 ± 9.59	0.388	0.040	1.088	0.009 ^†^
Age < 55 years old, women (*n* = 4180)										
	BMI	23.67 ± 2.96	23.68 ± 2.96	0.810	23.64 ± 2.99	23.66 ± 3.02	0.340	0.700	−0.005	0.967
	SBP	121.52 ± 15.17	121.34 ± 14.18	0.745	119.43 ± 14.81	119.79 ± 14.50	0.162	0.244	0.391	0.262
	DBP	75.90 ± 9.91	75.92 ± 9.28	0.958	74.81 ± 10.16	74.81 ± 9.81	0.992	0.263	−0.041	0.865
Age ≥ 55 years old, men (*n* = 1120)										
	BMI	24.76 ± 2.58	24.63 ± 2.66	0.139	24.85 ± 2.72	24.75 ± 2.74	0.024	0.758	−0.074	0.708
	SBP	126.48 ± 13.73	125.26 ± 12.00	0.236	128.92 ± 15.70	127.42 ± 14.65	0.011 *	0.909	−1.473	0.063
	DBP	79.13 ± 9.77	77.94 ± 8.40	0.011 *	80.17 ± 10.06	78.55 ± 9.51	<0.001 *	0.585	0.263	0.615
Age ≥ 55 years old, women (*n* = 4080)										
	BMI	24.45 ± 3.00	24.25 ± 3.10	<0.001 *	24.45 ± 3.06	24.41 ± 3.13	0.121	0.015 ^†^	0.007	0.953
	SBP	125.20 ± 15.53	125.02 ± 15.27	0.757	125.45 ± 16.47	125.40 ± 15.74	0.868	0.561	−0.715	0.092
	DBP	77.69 ± 10.00	76.69 ± 9.50	0.011 *	77.07 ± 10.25	76.42 ± 9.88	<0.001 *	0.464	0.779	0.004 ^†^

Abbreviations: BMI, body mass index, kg/m^2^; CCI, Charlson comorbidity index; EV, estimated value; SBP, systolic blood pressure; DBP, diastolic blood pressure; * paired *t*-test, significance at *p* < 0.05/3; ^†^ linear mixed model, significance at *p* < 0.053; ^‡^ interaction effects between time and group; ^§^ estimated value of linear mixed model for thyroid cancer II group based on the comparison II group; ^¶^ fixed effects were age, sex, income, region of residence, thyroid cancer, and time of measurement. Random effects were BMI, systolic blood pressure, diastolic blood pressure, fasting blood glucose, total cholesterol, smoking, alcohol consumption, and CCI scores.

## Data Availability

Release of the data by the authors is not legally allowed. Data in this study are available on the database of NHIS-HEALS in https://www.nhis.or.kr/ (assessed on 5 April 2021). NHIS permits access to all of these data via download for any researcher who promises to follow the research ethics.

## Supplementary Materials

The following supporting information can be downloaded at: https://www.mdpi.com/article/10.3390/ijerph19116753/s1.
