# Supplementary material for: Association between Thyroid Cancer and Weight Change: A Longitudinal Follow-Up Study
Source: Int J Environ Res Public Health. 2022 May 31;19(11):6753. doi: 10.3390/ijerph19116753 (PMC9180614; doi:10.3390/ijerph19116753)
Supplement: Supplementary file 1 [file ijerph-19-06753-s001.zip › ijerph-1686280-supplementary.pdf]

S1 description the statistical model of this study.

The pre- and postintervention values based on the index date were compared using paired t-tests. To analyze the interaction and estimated value (EV), a linear mixed model was used. Age, sex, income, region of residence, thyroid cancer, and time of measurement were used as the independent variables and fixed effects. The random effects of BMI, systolic blood pressure, diastolic blood pressure, fasting blood glucose, total cholesterol, smoking, alcohol consumption, and CCI scores were assessed. A first-order autoregressive model was selected as the repeated covariance type, considering the correlation of each participant's iteration. The linear mixed model analysis is as follows.

$$Y_i = X_{i1}\beta_1 + \dots + X_{ip}\beta_p + Z_{i1}\mathrm{u}_i + \dots + Z_{i\mathrm{q}}\mathrm{u}_q + e_i, \text{for all } i = 1, \dots, n$$

where $Y = (Y_1, \dots, Y_n)'$, X the $n \times p$ matrix of covariates with fixed effects $\beta = (\beta_1, \dots, \beta_p)'$, Z the $n \times q$ matrix of covariates with random effects $\mathrm{u} = \left(u_1, \dots, u_q\right)' \sim \mathrm{N}(0, \tau I_q)$, and the residual error vector $e = (e_1, \dots, e_n)' \sim \mathrm{N}(0, \tau I_n)$.

Table S2 Difference in mean values of BMI and blood pressure between pre and 1-year post of thyroid cancer in thyroid cancer I and comparison I group according to obesity

Characteristics	Thyroid cancer I			Comparison I			Interaction‡	Linear mixed model¶	
	Previous (mean, SD)	Post 1yr (mean, SD)	P-value	Previous (mean, SD)	Post 1yr (mean, SD)	P-value	P-value	EV§	P-value
Underweight (n= 100)									
BMI	17.93 ± 0.50	18.69 ± 1.69	0.060	17.65 ± 0.76	18.09 ± 1.21	<0.001*	0.225	0.275	0.298
SBP	118.65 ± 18.37	121.80 ± 11.95	0.467	116.10 ± 16.09	116.44 ± 15.12	0.837	0.172	-0.843	0.757
DBP	75.60 ± 11.21	75.65 ± 8.54	0.986	71.93 ± 10.20	73.95 ± 9.89	0.115	0.190	2.602	0.137
Normal (n= 3,340)									
BMI	21.37 ± 1.18	21.49 ± 1.56	0.101*	21.35 ± 1.17	21.58 ± 1.57	<0.001*	0.063	0.010	0.869
SBP	120.52 ± 15.50	120.10 ± 14.48	0.477	118.97 ± 15.28	119.80 ± 14.63	0.004*	0.878	0.061	0.880
DBP	75.34 ± 10.16	74.80 ± 9.88	0.193	74.00 ± 10.19	74.57 ± 9.79	0.005*	0.240	0.571	0.035
Overweight (n= 2,855)									
BMI	23.97 ± 0.56	23.90 ± 1.42	0.220	23.97 ± 0.57	23.99 ± 1.30	0.444	0.149	-0.001	0.979
SBP	122.94 ± 14.72	122.73 ± 13.84	0.746	122.83 ± 15.25	123.13 ± 14.57	0.360	0.399	-0.335	0.460
DBP	76.74 ± 9.65	76.49 ± 9.83	0.601	76.44 ± 10.09	76.26 ± 9.61	0.420	0.506	0.322	0.281
Obese I (n= 3,310)									

BMI	26.76 ± 1.30	26.54 ± 1.83	<0.001*	26.70 ± 1.26	26.47 ± 1.72	<0.001*	0.697	0.066	0.315
SBP	127.42 ± 14.19	126.68 ± 13.28	0.221	127.51 ± 15.61	126.63 ± 14.79	0.006*	0.623	-0.035	0.935
DBP	79.43 ± 10.10	78.81 ± 8.66	0.160	79.51 ± 10.32	78.54 ± 9.65	<0.001*	0.356	-0.017	0.952
Obese II (n= 370)									
BMI	31.88 ± 2.72	31.18 ± 3.00	0.002*	31.76 ± 2.28	31.07 ± 2.45	<0.001*	0.921	0.192	0.545
SBP	133.09 ± 18.46	130.89 ± 14.22	0.255	131.00 ± 16.21	129.73 ± 15.13	0.234	0.680	0.052	0.971
DBP	82.91 ± 10.26	81.65 ± 9.49	0.347	81.12 ± 10.57	80.16 ± 9.64	0.185	0.902	0.878	0.323

Abbreviations: BMI, body mass index, kg/m^2; CCI, Charlson comorbidity index; EV, Estimated value; SBP, systolic blood pressure; DBP, diastolic blood pressure

* Paired t-test, Significance at P < 0.05/3

† Linear mixed model, Significance at P < 0.05/3

‡ Interaction effects between time and group.

§ Estimated value of linear mixed model for thyroid cancer I group based on the comparison I group.

¶ Fixed effects were age, sex, income, region of residence, thyroid cancer, and time of measurement. Random effects were BMI, systolic blood pressure, diastolic blood pressure, fasting blood glucose, total cholesterol, smoking, alcohol consumption, and CCI scores.

Table S3 Difference in mean values of BMI and blood pressure between pre and 2-year post of thyroid cancer in thyroid cancer II and II group according to obesity

Characteristics	Thyroid cancer II			Comparison II			Interaction‡	Linear mixed model¶	
	Previous (mean, SD)	Post 2yr (mean, SD)	P-value	Previous (mean, SD)	Post 2yr (mean, SD)	P-value	P-value	EV§	P-value
Underweight (n = 155)									
BMI	17.80 ± 0.56	18.78 ± 2.07	0.008*	17.70 ± 0.75	18.12 ± 1.24	<0.001*	0.038†	0.081	0.713
SBP	117.06 ± 14.81	120.19 ± 16.97	0.370	115.39 ± 14.42	115.09 ± 13.42	0.812	0.461	3.995	0.023†
DBP	71.77 ± 8.50	73.16 ± 11.03	0.528	72.34 ± 9.69	72.18 ± 10.43	0.863	0.862	-1.418	0.240
Normal (n = 3,625)									
BMI	21.44 ± 1.10	21.62 ± 1.61	<0.001*	21.42 ± 1.09	21.65 ± 1.58	<0.001*	0.359	0.026	0.644
SBP	120.72 ± 15.76	120.89 ± 14.49	0.774	119.57 ± 15.43	119.66 ± 14.97	0.754	0.860	0.139	0.728
DBP	75.38 ± 10.03	75.13 ± 9.47	0.524	74.35 ± 10.23	74.12 ± 9.66	0.246	0.979	0.464	0.081
Overweight (n = 3,155)									
BMI	23.96 ± 0.57	23.85 ± 1.34	0.029	23.96 ± 0.57	23.96 ± 1.32	0.722	0.052	-0.003	0.950
SBP	123.48 ± 15.13	123.13 ± 14.10	0.588	123.12 ± 14.64	123.73 ± 14.78	0.052	0.902	-0.586	0.172
DBP	77.59 ± 9.82	76.55 ± 9.22	0.012*	76.70 ± 9.73	76.52 ± 9.86	0.408	0.313	0.762	0.006†
Obese I (n = 3,435)									

BMI	26.74 ± 1.28	26.47 ± 1.82	<0.001*	26.74 ± 1.28	26.51 ± 1.81	<0.001*	0.569	0.010	0.877
SBP	127.07 ± 13.69	125.96 ± 13.80	0.063	127.19 ± 15.81	126.67 ± 14.90	0.114	0.715	-0.532	0.222
DBP	79.49 ± 9.88	78.26 ± 8.98	0.004*	79.31 ± 10.27	78.43 ± 9.60	<0.001*	0.953	0.348	0.231
Obese II (n = 440)									
BMI	31.64 ± 1.71	31.06 ± 2.43	0.004*	32.00 ± 2.04	31.21 ± 2.88	<0.001*	0.436	-0.315	0.267
SBP	127.64 ± 15.39	128.15 ± 14.25	0.811	131.92 ± 16.34	130.55 ± 15.51	0.158	0.953	-2.457	0.073
DBP	79.70 ± 9.92	80.53 ± 8.43	0.537	81.25 ± 10.57	80.29 ± 9.65	0.163	0.401	0.345	0.700

Abbreviations: BMI, body mass index, kg/m^2; CCI, Charlson comorbidity index; EV, Estimated value; SBP, systolic blood pressure; DBP, diastolic blood pressure

* Paired t-test, Significance at P < 0.05/3

† Linear mixed model, Significance at P < 0.05/3

‡ Interaction effects between time and group.

§ Estimated value of linear mixed model for thyroid cancer II group based on the comparison II group.

¶ Fixed effects were age, sex, income, region of residence, thyroid cancer, and time of measurement. Random effects were BMI, systolic bloo d pressure, diastolic blood pressure, fasting blood glucose, total cholesterol, smoking, alcohol consumption, and CCI scores.
